# Autoimmune Polyglandular Syndrome Type 2: A Rare Condition in Childhood

**DOI:** 10.4274/jcrpe.1394

**Published:** 2015-03-05

**Authors:** Heves Kırmızıbekmez, Rahime Gül Yeşiltepe Mutlu, Nafiye Demirkıran Urgancı, Ayşe Öner

**Affiliations:** 1 Zeynep Kamil Obstetrics and Children Education and Research Hospital, Clinic of Pediatric Endocrinology, İstanbul, Turkey; 2 Şişli Etfal Education and Research Hospital, Clinic of Pediatric Gastroenterology, İstanbul, Turkey; 3 Academic Hospital, Clinic of Pediatric Nephrology and Rheumatology, İstanbul, Turkey

**Keywords:** autoimmune, polyendocrinopathy, management, children

## Abstract

Autoimmune polyglandular syndrome type 2 is defined as the occurrence of Addison’s disease concomitantly with autoimmune thyroid disease and/or type 1 diabetes mellitus. An 11-year-old boy with Hashimoto’s disease, Addison’s disease, celiac disease and Langerhans islet cell autoimmunity is described in this case report. Treatment of an endocrine disease may also trigger the onset of another endocrine disease. This case report underlines the importance of early recognition and treatment of critical endocrine diseases as well as the necessity to investigate pediatric patients with autoimmune diseases for coexisting conditions. Furthermore, the role of psychological stress as an inducer of autoimmunity was also discussed.

## INTRODUCTION

Autoimmune adrenal insufficiency (autoimmune Addison’s disease) is caused by destruction of the adrenal cortex by cell-mediated immune mechanisms. Addison’s disease may also present in the context of autoimmune polyendocrinopathy syndromes. The occurrence of Addison’s disease with autoimmune thyroid disease and/or type 1 diabetes mellitus is known as autoimmune polyglandular syndrome type 2 (APS-2) (1). The syndrome is associated with HLA-DR3 and/or HLA-DR4 haplotypes. The pattern of inheritance is autosomal dominant and the clinical expression is variable (2,3). APS usually presents in the third to fourth decades of life, with a highest prevalence in middle-aged women (4). The most frequent clinical combination of APS-2 is Addison’s disease and Hashimoto’s disease. Adrenal insufficiency has been reported in 5.3% of patients with Hashimoto’s disease (5). Two to 4% of autoimmune thyroid disease patients have concomitant celiac disease (6).

APS-2 is extremely rare in childhood and data on pediatric patients is limited to individual case reports. In this present report, a prepubertal male patient with Hashimoto’s disease, Addison’s disease, celiac disease and positive autoimmunity for type 1 diabetes is described. The need to investigate pediatric patients with organ-specific autoimmune diseases for coexisting conditions is also stressed.

## CASE REPORT

An 11-year-old boy was admitted to our clinic with complaints of fatigue, anorexia and weight loss which developed in the past eight months. He also had a history of severe psychological stress prior to the symptoms. Three months before the beginning of symptoms he had driven his father’s car without permission and crashed the car. There was not any physical injury in that accident, but the patient experienced serious psychological distress with the feeling of guilt. His parents also describe him as a sensitive, overly cautious and anxious child. His thyroid stimulating hormone level was found as 12.4 mIU/mL with positive thyroid antibodies and he was diagnosed as a case of subclinical hypothyroidism due to “Hashimoto thyroiditis”. He was prescribed 50 µg/day L-thyroxine (L-T4) therapy. However, despite this treatment, clinical deterioration occurred. He lost 8 kg in body weight within three months.

Physical examination at presentation revealed a blood pressure level of 90/40 mmHg and presence of grade 1 goitre. Height was 141 cm and weight was 32 kg (standard deviation score values were 0.54 and -0.74, respectively). Pubertal findings were consistent with Tanner stage 1. The patient had a suntanned appearance, but there was no marked hyperpigmentation in the skin or mucosa. A low cortisol level accompanied by a high adrenocorticotropic hormone (ACTH) level confirmed presence of primary adrenal insufficiency. Laboratory findings at referral and follow-up visit are presented in Table 1. Thyroid ultrasonography revealed a heterogeneous pattern of the parenchyma with no nodular lesion. The electrolyte imbalance was treated with saline infusion. A diagnosis of adrenal insufficiency was considered and treatment with glucocorticoids (10 mg/m2/day hydrocortisone) and mineralocorticoids (0.1 mg/m2/day fludrocortisone) was started. L-T4 treatment was ceased and started again as soon as the clinical findings improved. A very long-chain fatty acid profile was normal and adrenoleucodystrophia was excluded. Investigation for other associating autoimmune endocrine diseases revealed positive findings for anti-glutamate dehydrogenase, anti-islet cell, anti-gliadin and anti-endomysium antibodies. The oral glucose tolerance test revealed a normal fasting blood glucose level and normal glucose tolerance values (Table 1). Endoscopic biopsy revealed villous atrophy, proliferation of the crypts and increased lymphocytic infiltration, confirming the presence of gluten-sensitive enteropathy (celiac disease).

## DISCUSSION

Our patient was an adolescent boy with a diagnosis of APS-2, a condition which is quite rare in childhood. APS-2 usually occurs in patients 30 to 40 years of age and women are affected three times more than men (4). Acquired adrenocortical insufficiency is also uncommon in children and since symptoms like fatigue, anorexia, nausea, weight loss and cognitive disorders are nonspecific and may be related with other conditions such as hypothyroidism, infections and other systemic diseases, adrenocortical insufficiency at this stage can easily be underdiagnosed in pediatric practice. Hypotension, hypoglycemia and hyponatremia are fairly late manifestations (7). Skin hyperpigmentation may be a clue for primary adrenal insufficiency, but it may not be remarkable especially in persons who already have a suntanned skin. Our patient did not have a significant hyperpigmentation either in his skin or in his oral mucosa despite the very high ACTH level. Weight loss is a crucial finding, since it is not expected in hypothyroidism. The complaints of our patient were exacerbated after prescription of L-T4, possibly due to the enhancing effect of T4 on hepatic corticosteroid metabolism. The small increase observed in hemoglobin A1c level after replacement therapy suggested the possibility of subclinical hypoglycemic attacks before the diagnosis of adrenal insufficiency.

After the diagnosis of adrenal insufficiency, investigation for possible coexisting diseases revealed presence of celiac disease and positive autoimmunity for type 1 diabetes. The majority of these patients have an atypical or silent type of celiac disease. This is the main reason why it is often underdiagnosed. Early application of a gluten-free diet and lifelong adherence to this treatment decreases the risk of complications in celiac disease (8). Betterle et al (7) reported that clinically overt disorders are considered to be the tip of the autoimmune iceberg since latent forms are much more frequent. This patient might develop type 1 diabetes during the follow-up period. These conditions are designated as “incomplete APS”. Pancreatic antibodies are present in about 6-20% of patients with Addison’s disease who have no overt type 1 diabetes. However, it is not well known whether or when these patients will develop overt disease (7).

Our patient also had a history of psychological stress followed by nonspecific complaints (e.g. anorexia, fatigue, anxiety and altered school performance) suggesting post-traumatic stress disorder. Recent reports indicate the possible role of psychological stress and the role of the major stress-related hormones in the pathogenesis of autoimmune diseases (9). The majority (up to 80%) of patients were reported to suffer from emotional stress before disease onset (10).

In conclusion, we report this patient with APS-2, a rare condition in childhood. Replacement of thyroid hormone may induce exacerbation of a silent adrenal insufficiency. Further investigations should be performed if the patient’s condition deteriorates rather than improves following the hormone replacement therapy. We also suggest that the long-term follow-up of these patients is very important, as incomplete forms are expected to be more common in childhood.

## Figures and Tables

**Table 1:**
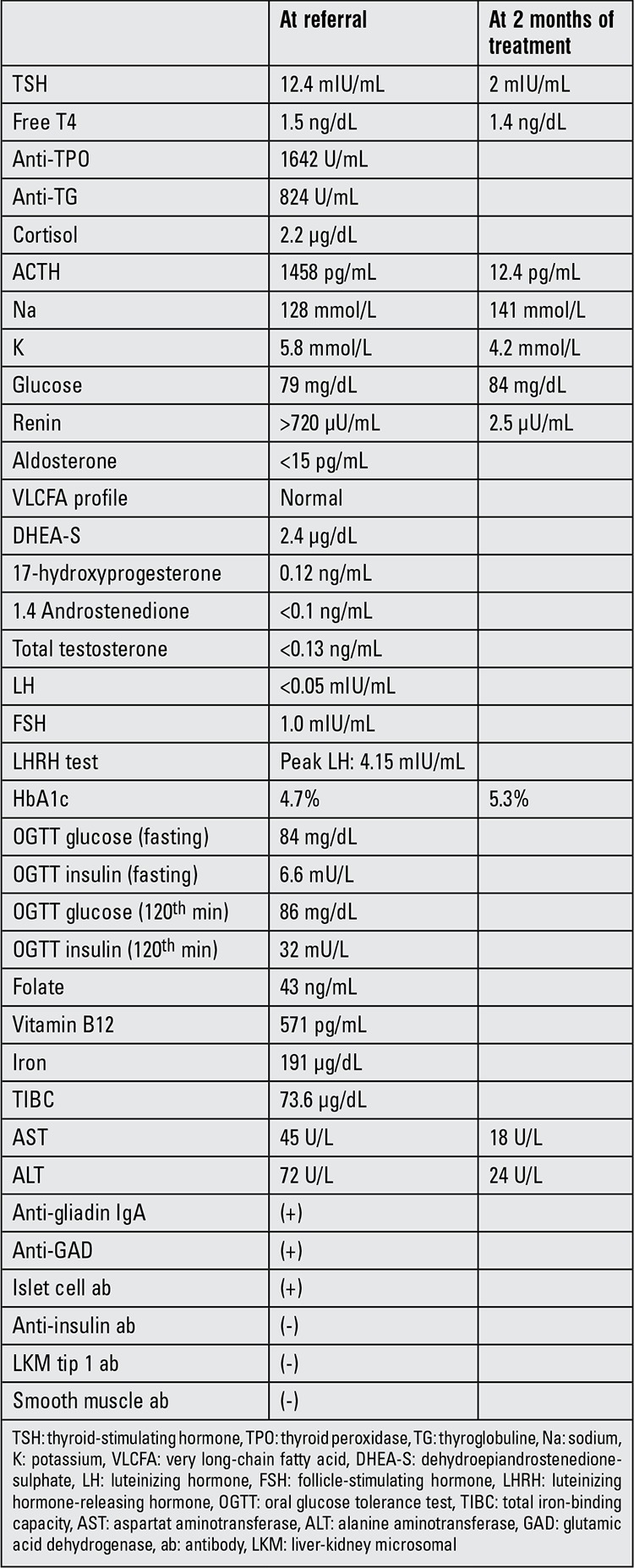
Biochemical findings of the patient at referral and at 2 months of treatment
